# Directional selection to improve the sterile insect technique: Survival and sexual performance of desiccation resistant *Anastrepha ludens* strains

**DOI:** 10.1111/eva.12506

**Published:** 2017-07-06

**Authors:** Marco T. Tejeda, José Arredondo‐Gordillo, Dina Orozco‐Dávila, Luis Quintero‐Fong, Francisco Díaz‐Fleischer

**Affiliations:** ^1^ INBIOTECA Universidad Veracruzana Xalapa México; ^2^ Departamento de Filtrado Genético Programa Moscamed acuerdo SAGARPA‐IICA Metapa de Domínguez México; ^3^ Departamento de Biología, Ecología y Comportamiento, Desarrollo de Métodos Programa Moscafrut Acuerdo SAGARPA‐IICA Metapa de Domínguez México; ^4^ Subdirección de Producción Programa Moscafrut Acuerdo SAGARPA‐IICA Metapa de Domínguez México; ^5^ Departamento de Validación Tecnológica, Desarrollo de Métodos Programa Moscafrut Acuerdo SAGARPA‐IICA Metapa de Domínguez México

**Keywords:** area wide integrated pest management, experimental evolution, Mexican fruit fly, sexual isolation, Tephritidae

## Abstract

The sterile insect technique (SIT) is an effective, environmentally friendly method for insect control whose success depends on the sexual performance and survival of sterile males. These two parameters are influenced by environmental conditions of target areas, and releasing insects with a higher tolerance to stressful environments can improve SIT efficiency. Directional selection can be used to produce insect strains with higher tolerance to extreme environmental conditions, such as low humidity, for extended periods. We evaluated, under field cage conditions, the sexual competitiveness, sexual compatibility, and survival of strains of *Anastrepha ludens* (Loew) selected for desiccation resistance to determine the value of directional selection as a possible approach to enhance SIT efficiency. Fly strains (selected and unselected and those mass‐reared) were exposed to stressful conditions of low humidity and food and water deprivation for 24 hr before test. As a control, mild conditions without the stressors were used. No differences in sexual competitiveness and sexual compatibility between selected, nonselected, and mass‐reared strains were observed when previously exposed to mild conditions. Thus, selection for desiccation resistance does not modified negatively the sexual performance. However, when insects were exposed to stressful conditions, males of selected strains sexually outperform mass‐reared males. Additionally, selected strains presented higher survival than mass‐reared flies. The approach to integrate directional selection with other technologies in the SIT as well as the implications of using a desiccation‐selected strain in the current pest management program is discussed.

## 1 INTRODUCTION

The sterile insect technique (SIT) is one of the most effective, specific, and environmentally friendly method of insect control (Dyck, Hendrichs, & Robinson, 2005; Klassen, 2005). SIT bases in the mass production of males that are sterilized and released in the field, where they compete with wild males for wild female mates. The success of SIT depends on the ability of sterile males to compete for mates with wild males. Copulations between sterile males and fertile wild females must results in the production of no offspring and the consequent reduction in insect pest population on target areas (Knipling, 1955, 1979). Additionally, SIT entails maintaining a high ratio of sterile to fertile males on target areas to increase the probability of sterile male/wild female copulations (Barclay, 2005; Knipling, 1968). To efficiently sustain the population of sterile males in the field, a balance between the quantity of insect released and the frequency of releases needs to be found (Hendrichs, Vreysen, Enkerlin, & Cayol, 2005). As this balance largely depends on sterile males survival, it has been noted that a slight increment in daily insect survival has a greater impact in SIT efficiency than a great increment in the number of individuals released (Liedo, Oropeza, & Carey, 2010). Thus, the survival of these sterile male is crucial to uphold the sterile to fertile male ratio over time (Calkins & Parker, 2005; Lance & McInnis, 2005; Vreysen, 2005).

Relative humidity and water availability are factors that affect the survival and reproduction of organisms (Begon, Townsend, & Harper, 2006). Because of their small size and large area to volume ratio, insects are especially susceptible to water loss (Price, 1997), a condition that leads to desiccation. For their ecological relevance, desiccation resistance has been earmarked as a life history character capable of determine the distribution, permanence, and abundance of species (Kellermann, van Heerwaarden, Sgrö, & Hoffmann, 2009; Marron, Markow, Kain, & Gibbs, 2003). For insect pests like fruit flies, their innate capacity to maintain a population in a given place is related to abiotic factors like relative humidity, and to intrinsic factors that constrains or shapes the response to environmental conditions (Meats, 1989a, 1989b). Thus, desiccation resistance can also be classified as a relevant ecological trait for pest management strategies. When using SIT as a control method, it is important to take into account traits like desiccation resistance because several release areas imposes stressful conditions (Rempoulakis & Nestel, 2012; Vanoye‐Eligio, Mora‐Olivo, Gaona‐García, Reyes‐Zepeda, & Rocandio‐Rodríguez, 2017) that can affect the performance of laboratory insects. Furthermore, the operative importance of traits like desiccation resistance is accentuated when consider that rearing insects in the laboratory leads to a decrease in their stress resistance (Hoffmann, Hallas, Sinclair, & Partridge, 2001; Weldon, Yap, & Taylor, 2013) and that wild populations can present some degree of local adaptation to stressful conditions (Gilchrist et al., 2008; Karan et al., 1998).

The SIT has proved to be a specific and ecologically friendly method for insect pest control; however, a reduced ability to survive in the field of mass‐reared insects is a pitfall that needs attention (Sørensen, Addison, & Terblanche, 2012; Terblanche & Chown, 2007). In the case of desiccation resistance, several genes are involved in their expression (Foley & Telonis‐Scott, 2011; Kang, Aggarwal, Rashkovetsky, Korol, & Michalak, 2016; Kawano et al., 2010; Sinclair, Gibbs, & Roberts, 2007; Sørensen, Nielsen, & Loeschcke, 2007; Telonis‐Scott & Hoffmann, 2003; Telonis‐Scott, Gane, DeGaris, Sgrö, & Hoffmann, 2011). Thus, directional selection stands as a proper strategy to increase desiccation resistance of strains that already have adapted to the mass rearing environment (Falconer & Mackay, 1996; Hendry et al., 2011; Lommen, de Jong, & Pannebakker, 2017). In the model insect *Drosophila melanogaster* [Meigen], experimental evidence has shown that selection for desiccation and starvation has a positive impact on longevity and confers resistance for other stressors (Archer, Phelan, Beckman, & Rose, 2003; Bubliy & Loeschcke, 2005; Harshman, Hoffmann, & Clark, 1999; Hoffmann & Parsons, 1993; Rose, Vu, Park, & Graves, 1992; Telonis‐Scott, Guthridge, & Hoffmann, 2006). Therefore, selection of stress resistance stands as a promising application to enhance SIT efficiency.

For the tephritid *Anastrepha ludens* [Loew], a set of desiccation‐resistant selected strains has been established recently. These selected strains show twice of the resistance to desiccation of that observed in control populations after ten generations of selection (Tejeda et al., 2016). Similarly to *Drosophila*, the desiccation resistance strains of *A. ludens* exhibit an extended longevity in both sexes as a correlation response to selection, a positive character for SIT (Tejeda et al., 2016). However, there exists the concern that directional selection modifies, directly or indirectly, sexual behavior and propitiate assortative matings reducing the effectiveness of SIT (Cayol, 1999; McInnis, Shelly, & Komatsu, 2002; Miyatake, 1998; Sørensen et al., 2012). For example, Miyatake (1997, 2002) observed that the selection for development period altered the time of mating and circadian rhythm of *Bactrocera cucurbitae* [Coquillett] and that this changes derives in a premating isolation between strains (Miyatake et al., 2002). However, evidence of interaction of desiccation resistance with mating success is somewhat variable. In *Drosophila*, a positive interaction has been reported in one study (Gefen & Gibbs, 2009) but not in others (Hoffmann & Parsons, 1993; Kwan & Rundle, 2010). Because matings are intimately linked with the suppression of pest populations in SIT (Calkins & Parker, 2005; Itô & Yamamura, 2005; Lance & McInnis, 2005; Vreysen, 2005), detailed information about the ability of desiccation‐selected strain to sexually compete with wild populations for mates is mandatory and sorely needed for operative pest management programs.

In this study, we evaluated the sexual performance of a desiccation‐resistant selected strain of *A. ludens*. This species is a polyphagous, multivoltine, highly plastic fruit fly that can be found in a wide range of environments, from the semiarid lands of south of Texas to the tropical rain forests of Costa Rica (Hernández‐Ortiz & Pérez‐Alonso, 1993). It is considered a pest of several economically important fruit crops such as citrus and mangoes (Norrbom & Foote, 1989; Norrbom & Kim, 1988). To protect the fruit industry, *A. ludens* are subject to an area‐wide integrated pest management program where more than 120 million flies are produced, sterilized, and released per week (Gutiérrez‐Ruelas, 2010). The large scale and sustained application of SIT has been a key factor to maintain and expand the pest free areas (Liedo, 2016), and importantly, their also had gathered data of how SIT efficiency could be enhanced. While male life expectancy in the laboratory has been observed between 39 and 130 days (Carey et al., 2005, 2008; Liedo, Carey, Celedonio, & Guillen, 1992; Tejeda et al., 2016), in field evaluations, where variable environmental conditions prevails, life expectancy oscillate around 2–10 days (Flores et al., 2015; Hernández, Orozco, Breceda, & Domínguez, 2007; Thomas & Loera‐Gallardo, 1998; Utgés, Vilardi, Oropeza, Toledo, & Liedo, 2013). The contrasting male life expectancies between laboratory and field indicate the possibility that sterile males are exposed to stress once released in the field. Thus, desiccation‐resistance males may represent a technical advantage in stressful environments. Based on this, we aimed at three objectives: (i) determining the effect of selection upon the ability to compete for mates (selected strain vs. control [no‐selected] strain), (ii) ascertaining the competitiveness and compatibility of selected strains and compare it with that of the currently released strain (selected strain vs. mass‐reared strain), and (iii) comparing the survival and competitiveness between selected and mass‐reared strains when facing a stressful environment.

## 2 METHODS

### 2.1 Insects

Four groups of *A. ludens* flies were used: (i) selected, (ii) control (unselected), (iii) mass‐reared (Moscafrut), and (iv) wild flies. Selected flies (i) were obtained from five populations, D1–D5, where the 10% of the population had survived a desiccation stress (20%–30% relative humidity). Control flies (ii) were obtained of five populations, C1–C5, in which the desiccation stress was absent and flies mated randomly each generation. Both strains were established from the mass‐reared flies of the Moscafrut factory (Tejeda et al., 2016). At the time of the study, flies were of the 13 and 14 generations of the evolutive experiment. A pool of flies was generated for each evolutive regimen (selected or control) using pupa of five independent populations (D1–D5 and C1–C5, respectively). For the third group, Moscafrut (iii), we used individuals of the mass rearing process of Moscafrut facilities at Metapa de Dominguez, Chiapas, Mexico (Domínguez, Artiaga‐López, Solís, & Hernández, 2010). Finally, Wild flies (iv) were obtained of naturally infested fruits of *Citrus aurantium* L. (bitter orange) collected in the Soconusco region of Chiapas, Mexico. Infested fruits were dissected in the laboratory, and mature larva were allowed to pupate in moisten vermiculite.

### 2.2 Rearing and handling

To simulate the conditions in which the insects are used on the SIT, laboratory insects (selected, control, Moscafrut) were exposed, in hypoxia and 48 hr before emergence, to the standard sterilization dose of 80 Gy using a ^60^Co irradiator (model GB‐127, Nordion International Inc., Ottawa, ON, Canada).

Once emerged, we separated the insects by sex. Each sex was kept in 30x30x30 cm cubic wood framed‐mesh covered cages. Insects were provided with water and standard food (sugar and hydrolyzed yeast [MP 126 Biomedicals; LLC, Santa Ana, CA, USA] in a 3:1 ratio) and maintained in 12:12 light–dark photoperiod, 60%–80% relative humidity, and 25 ± 2°C temperature. Laboratory insects were maintained in a density of 150–200 individuals per cage. To avoid over‐stressing the wild flies in the laboratory, wild flies were kept at a density of 100 individuals per cage.

The insects were properly marked according to treatment a day before field cage tests by attaching a small printed label that had a number or letter on the thorax (Food and Agriculture Organization of the United Nations/International Atomic Energy Agency/United States Department of Agriculture [FAO/IAEA/USDA], 2014). This type of mark does not interfere with mate choice (McInnis, Rendon, & Komatsu, 2002; Meza, Díaz Fleischer, & Orozco, 2005). On the experimental day, marked insects were transferred per sex and strain to cubic 15‐cm cages following the experimental protocols. This procedure was carried out to replace damaged insects, facilitate the transport and release. Wild insects were between 15 and 18 and laboratory insects between 11 and 14 days old, age at they reach sexual maturity (FAO/IAEA/USDA, 2014).

### 2.3 Field cages set up

All the evaluations of insect′s sexual behavior were performed under the seminatural conditions of walk‐in field cages. The field cage used for quality control tests consisted of a cylindrical grid of 3 m diameter and 2 m high with an artificial canopy made of host trees inside (Calkins & Webb, 1983). Although it is impossible to reproduce the exact characteristics of a cage, we recognized a priori two groups of field cages. In one group, potted trees of orange and mango were arranged around the inner circumference and on the center of the cage (Díaz‐Fleischer & Arredondo, 2011; Meza‐Hernández & Díaz‐Fleischer, 2006). In the other group, the host tree was planted in the ground at the center of the cage, forming a canopy 1.5 m above the ground that cover approximately 80% of the top surface of the cage. Taking into account the above and to avoid bias, we balanced the possible effect of cage: all treatments were conducted on each field cage type, and treatments were alternated within cages each trial.

Bioassays started at 16:00 by releasing males first to allow them to acclimatize and set territories. Females were released 1 hr later. As *A. ludens* flies copulate at dusk (Aluja, Piñero, Jácome, Díaz‐Fleischer, & Sivinski, 1999), they were constantly monitored until 20:00 hr. All mating pairs were individually collected in petri dishes for treatment identification.

### 2.4 Experimental designs

#### 2.4.1 Effect of selection on competitiveness

To evaluate the effect of selection on male sexual competitiveness, 25 selected males and 25 control (unselected) males were released into each field cage where they competed for mating with 25 wild females. Fifteen replicas were carried out.

#### 2.4.2 Compatibility of selected strain

The compatibility of the selected desiccation resistant strain with wild population was compared to Moscafrut flies. The compatibility test consisted of releasing, in a field cage, the laboratory strain (females and males) and the wild strain (females and males) (Cayol, Vilardi, Rial, & Vera, 1999; FAO/IAEA/USDA, 2014). With such a scheme, we established two treatments. In the first treatment, 20 selected females and 20 selected males were released together with 20 wild males and 20 wild females. The second treatment consisted of 20 females and 20 males of the Moscafrut mass‐reared strain released together with 20 wild males and 20 wild females. A total of 28 experimental units (cages) were observed (2 treatments × 14 replicates).

#### 2.4.3 Survival and competitiveness under stressful conditions

To assess the potential benefits of releasing a desiccation resistant phenotype, the sexual competitiveness between the selected and Moscafrut mass‐reared males was evaluated under two experimental scenarios. First, released insects were exposed to a stressful environment of low relative humidity (30%–40%) environment and no water or food was available 24 hr before field cage bioassay. Second, mild environment, the insects were plenty of water and food and were exposed to a relative humidity (60%–80%) before test.

Low relative humidity environment was attained by the introduction of a desiccator substance (silica gel) inside a cubic Plexiglas cage of 25 cm enclosed with a plastic, 30 males were placed inside, and the cage represented the experimental unit (Tejeda et al., 2014). The silica gel (150 g) was distributed in three plastic containers covered with a mesh lid to avoid contact with the flies. For the “mild” (un‐stressful) environment treatment, the cages were kept under laboratory environmental condition (60%–80% relative humidity and 25±2°C temperature) and not enclosed with a plastic cover, and additional water and food was provided ad libitum. Moscafrut and selected males were exposed to treatments in separate cages. Survival of individuals was recorded for each plexiglas cage after 24 hr of exposure. We assayed three different cohorts of each strain for a total of 54 experimental units.

After environmental treatment, we released 25 Moscafrut males, 25 selected for desiccation males, and 25 wild (nonstressed) females inside a field cage to measure competitiveness. This bioassay was repeated for each environmental treatment. A total of eight mild treatment and six stressful treatment field cages were observed.

### 2.5 Indexes of sexual performance

Following practices for mating behavior analysis of tephritid fruit flies of economic importance (FAO/IAEA/USDA, 2014), male sexual competitiveness and strain sexual compatibility were calculated using the indexes of frequencies per mating type (Cayol et al., 1999; McInnis, Lance, & Jackson, 1996; Meza‐Hernández & Díaz‐Fleischer, 2006). Four mating type categories were possible: *SS, SW, WS, WW*, where first letter indicates male, second letter females, “*S*” sterile, and “*W*” wild.

The relative sterility index (RSI) was used to compare male sexual competitiveness for their capability to express the proportion of matings obtained for each male in competition. Values of RSI can vary from 0 to 1, where 0 indicates that all of the wild females that mated in the cage mated with wild males, 1 indicates that they all mated with sterile males, and .5 indicates that half mated with sterile males and half with wild males and that sterile males are equally competitive with wild males.

The sexual compatibility between strains was evaluated by the index of sexual isolation (ISI) which values range from −1 (negative assortative mating, all heterotypic matings) to 0 (random mating, full sexual compatibility) to +1 (positive assortative mating, i.e., total sexual isolation). Additionally, the male and female relative performance indexes (MRPI and FRPI) were computed to account from mating differences between sexes on the compatibility test.

$$\text{RSI}=\frac{SW}{SW+WW}$$

$$\text{ISI}=\frac{SS+WW-SW-WS}{SS+WW+SW+WS}$$

$$\text{MRPI}=\frac{SS+SW-WS-WW}{SS+WW+SW+WS}$$

$$\text{FRPI}=\frac{SS+WS-SW-WW}{SS+WW+SW+WS}$$

### 2.6 Data analysis

Once indexes of competitiveness and compatibility were computed for each field cage (experimental unit), a one‐sample Student′s *t* test was performed to determine whether the index (RSI, ISI, MRPI, or FRPI) differs significantly from the theoretical value expected for equal competitiveness or random mating. Additionally, a Welch *t* test was performed to determine whether the indexes statistically differ between desiccation‐selected and Moscafrut mass‐reared strains. For the experimental design (2.4.1) where selected males competed with control (unselected) males, the RSI was calculated by considering the selected strain as the “*S*” and control strain as the “*W*” on the formula of the RSI.

The effect of a stressful environment on survival was analyzed using an ANOVA where survival was fitted as the dependent variable and environmental treatment, strain, and the interaction between them were fitted as predictors. Treatment comparisons were performed using the exact *F* test.

## 3 RESULTS

### 3.1 Effect of selection on competitiveness

**Figure 1:**
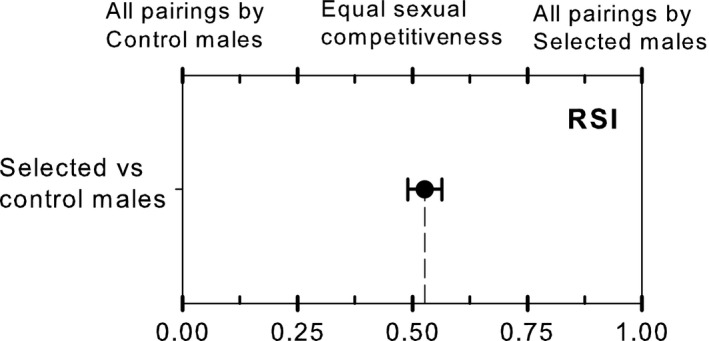
Effect of selection on sexual competitiveness. The relative sterility index (RSI) indicated that desiccation‐selected males secured a similar proportion of wild females mates when competed with control (unselected) males under field cage conditions. Dot and error bars indicate mean and standard error, respectively

Along the test of sexual competition between selected and control (unselected) males, we obtained, on average, 38.6% of possible mating pairs in the field cages, which is higher than the minimum 20% recommended for analysis by the FAO/IAEA/USDA (2014) and common for this species. Considering the selected strain as the laboratory material on the formula of RSI, we obtained a RSI of .53 ± .04 (mean ± SE), indicating that selected males secured, on average, 53% of the matings per cage. The computed RSI was not statistically different from the theoretical expected value of .5 where selected males equally compete with control males (one‐sample *t* test, *t*_13_ = 0.73, *p* = .48); thus, no significant differences on competitiveness were detected between selected and control strains (Figure 1).

### 3.2 Compatibility of selected strain

**Figure 2:**
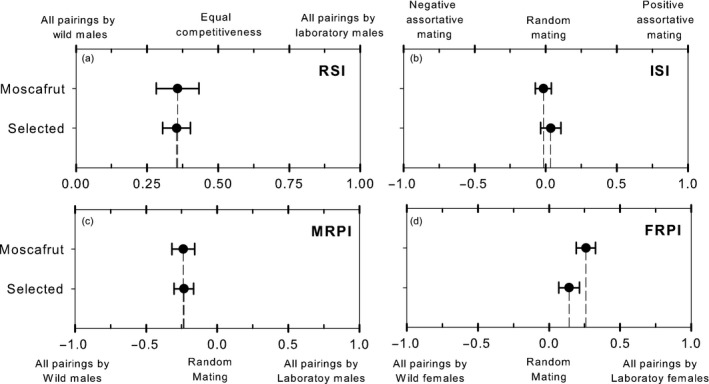
Sexual compatibility with wild population. Comparisons of the relative sterility index (RSI) (a), Index of sexual isolation (ISI) (b), Male relative performance index (MRPI) (c), and Female relative performance index (FRPI) (d) of Moscafrut and desiccation‐selected strains. For each index, the value obtained was not different between strains. Dot and error bars indicate mean and standard error, respectively

An average of 37% possible matings between Moscafrut mass‐reared strain and wild flies and 33% of possible matings between desiccation selected and wild flies were recorded in the field cages. In the presence of wild males, laboratory males secured about 35% of mates with wild females: RSI of Moscafrut mass‐reared strain = .36 ± .07 and RSI of selected strain = .35 ± .05. The statistical analysis pointed mixed results. While RSI of the Moscafrut strain was not significantly different from the expected on equal competitiveness between laboratory and wild males [theoretical value for equal competitiveness = .5] (*t*_11_ = 1.9, *p* = .08), statistical differences were recovered for the selected strain (*t*_13_ = 2.96, *p* = .01). However, the direct contrast of the competitiveness index between strains indicated that RSIs were not statistically different from each other (Welch Test, *t*_19.5_ = 0.03, *p* = .97), suggesting that males of both strains are equally competitive with wild males (Figure 2a).

In respect of the sexual compatibility between laboratory strains and wild flies, we found close to zero values on the ISI. We recovered an ISI of –.02 ± .06 for Moscafrut strain, while for selected strain, we recovered .04 ± .07. Statistical analysis indicated that ISI values were not significantly different from those expected for random mating [theoretical value for random mating = 0] (*t*_11_ = 0.3, *p* = .76 and *t*_13_ = .5, *p* = .63 for Moscafrut and selected strains, respectively). The direct contrast of the compatibility between strains indicated that the observed ISIs were not statistically different from each other (Welch Test, *t*_23.4_ = 0.57, *p* = .57), suggesting that both Moscafrut and desiccation‐selected strains are equally sexual compatible with the wild population (Figure 2b).

To obtain information of each sex performance, MRPI and FRPI were analyzed. We found negative values of MRPI on both Moscafrut (−.24 ± .08) and selected strains (−.23 ± .07). The analysis indicated that MRPI were statistically different from the theoretical value expected on equal participation in matings between wild and sterile males [0] (*t*_11_ = 9.2, *p* < .01 and *t*_13_ = 3.4, *p* < .01, for Moscafrut and selected strains, respectively), revealing that wild males were engaged in more mating pairs. The direct contrast indicated that MRPI were not different between laboratory strains (Welch Test, *t*_22.7_ = .04, *p* = .96), suggesting that selected and Moscafrut mass‐reared males presents equal mating propensity in the field cages (Figure 2c). Similar, positive values of FPRI were obtained for both Moscafrut (.26 ± .07) and desiccation‐selected (.14 ± .07) strains. The statistical analysis recovered mixed results. While significant differences from the theoretical expected for equal participation in matings between wild and sterile females [0] were recovered for Moscafrut strain (*t* test, *t*_11_ = 3.5, *p* < .01), the FRPI of selected lines adjusted to the theoretical expected (*t* test, *t*_13_ = 1.9, *p* = .07), indicating a relatively higher participation of sterile females on matings for the evaluation of the Moscafrut strain. However, the direct contrast of relative performance revealed no significant differences between FMRIs (Welch Test, *t*_23.9_ = 1.19, *p* = .24), suggesting that females of both, Moscafrut and desiccation‐selected strains, presented a similar mating propensity in the field cages (Figure 2d).

### 3.3 Survival and competitiveness under stressful conditions

**Figure 3:**
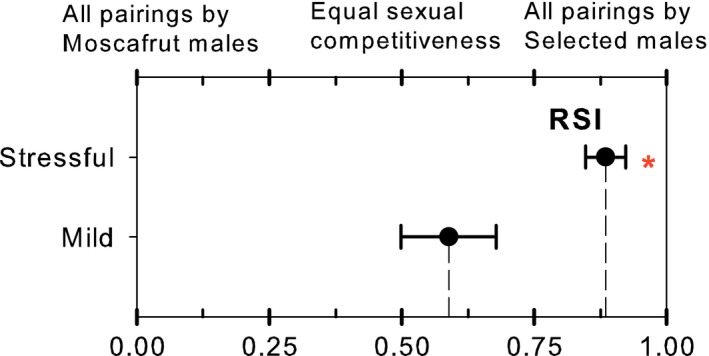
Competitiveness on stress. Comparison of the relative sterility index (RSI) of Moscafrut and desiccation‐selected males competing for wild females mates after mild and stressful environmental conditions. Dot and error bars indicate mean and standard error, respectively. For the stressful treatment *indicates significant deviation from the expected on equal sexual competitiveness

In the field cages where selected and Moscafrut mass‐reared males competed for wild female mates, we recovered 39% and 23% of possible matings for the mild (un‐stressful) and stressful environment treatment, respectively. Selected males secured 59% of matings on the mild treatment and 88% of matings on the stressful treatment. On the un‐stressful treatment, the competitiveness index (RSI = .59 ± .25) was not statistically different from the expected on equal competitiveness between males [.5] (*t*_7_ = 1, *p* = .36). On the stressful treatment however, the competitiveness index (RSI = .88 ± .09) was significantly different from the expected (*t*_5_ = 10.1, *p* < .001); indicating that males of the selected strain sexually outperform Moscafrut mass‐reared males when previously exposed to stressful conditions (Figure 3).

**Figure 4:**
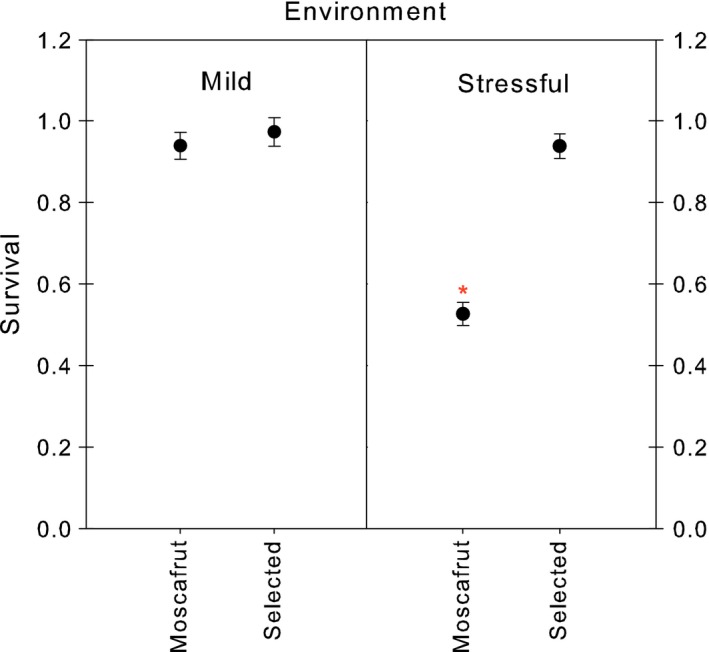
Comparative survival of Moscafrut and desiccation‐selected males in plexiglas cages under mild and stressful conditions. Dots and error bars indicate mean and standard error, respectively. Within environmental treatment *indicates significant differences between strains

The analysis of the effect of contrasting environments on survival indicated that strains were differently affected for the environmental conditions (Strain: treatment interaction *F*_1–48_ = 36, *p *< .001). While survival under mild un‐stressful conditions was not different between strains (*F*_1–48_ = 0.5, *p* = .48), the survival of the selected strain was significant higher that the Moscafrut mass‐reared strain when exposed to stressful conditions (*F*_1–48_ = 101, *p* < .0001) (Figure 4).

## 4 DISCUSSION

Desiccation resistance selection did not lead to precopulatory sexual isolation with the wild population of *A. ludens*. Evaluations in the field cages confirmed equivalent or superior sexual behavior parameters of the selected strain compared to those obtained for the strain currently used in the SIT program against *A*. *ludens* in México. The main advantage of the desiccation‐selected strain was observed when flies were exposed to adverse environmental conditions before competing for mating with wild females. Selected males presented higher survival and secured more mates with wild females than the Moscafrut male counterparts.

The estimated ISIs and RSIs values obtained in the three different experiments suggest total compatibility between the selected strain and the wild population of *A*. *ludens* studied here. Additionally, the MRPIs did not differ from the expected, indicating comparable male mating competitiveness of control, mass‐reared and selected flies. This pattern of sexual competitiveness, however, becomes favorable for selected flies when exposed to a harsh environment before sexual competition. Sexual advantage may be consequence of not only for the innate resistance to the low humidity conditions of selected flies but also for the higher levels of nutritional reserves that allow them to endure starvation (Tejeda et al., 2014, 2016). These results indicate that selection of desiccation resistance is a promising strategy to improve the efficiency of sterile males.

From a SIT application view, the extended survival of selected strain instantly translates into a positive impact on efficiency by reducing the frequency of releases needed to maintain a fixed number of sterile males in the field (Hendrichs et al., 2005; Liedo et al., 2010) and thus reducing the cost associated to release activities. Furthermore, in the long term, higher survival in the field also represents a reduction in the absolute quantity of sterile males needed to accomplish the pest control objective. Additionally, as selected males obtained more mating pairs, it can be expected than in adverse environments the release of resistant males will induce more sterility on the target population. In the case of *A. ludens,* the Campaign against Fruit Flies is currently using SIT in northern and central regions of Mexico (Gutiérrez‐Ruelas, 2010), where dry climate with an average annual precipitation lower than 600 mm and temperatures that can exceed 30°C predominates (Comisión Nacional del Agua [CONAGUA], 2015). Thus, if the field survival of the standard strain is compromised, the release of desiccation resistant males promises to be a more efficient strategy, leading to lesser frequency of releases, less sterile insects to be produced for the biofactory and more infertile matings in the field per released male.

Desiccation resistance has been repeatedly successful selected in *Drosophila* and *Anastrepha* (Archer et al., 2003; Bubliy & Loeschcke, 2005; Gefen, Marlon, & Gibbs, 2006; Hoffmann & Parsons, 1993; Rose et al., 1992; Telonis‐Scott et al., 2006; Tejeda et al., 2016) and apparently does not generates precopulatory sexual isolation (Kwan & Rundle, 2010). Therefore, it is likely that the extended survival and competitiveness showed by resistant insects can be replicated in other biological systems (e.g., Lommen et al., 2017) and thus, several pest management programs could benefits from the evolutionary approach. In addition, directional selection strategies for specific characters, like desiccation resistance, can be combined with other genetic strategies that improve SIT efficiency. For example, genetic sexing strains and genetically modified insects, both highly valuables for SIT, often present less than optimal performance for some biological parameters (Cayol et al., 1999; Meza, Nirmala, Zimowska, Zepeda‐Cisneros, & Handler, 2011; Meza et al., 2014). Performance on these strains can be improved with directional selection. However, as several of these strains were originated from a single or from very few individuals, a suitable approach could involve the selection on lines with richer genetic pool and the subsequent introgression of the character (cross of strains following by repeated backcrossing). From a practical point of view, candidate characters that potentially increase SIT efficiency include desiccation and/or temperature tolerance (Sørensen et al., 2012; Tejeda et al., 2014; Wang, Johnson, Daane, & Nadel, 2009), avoidance of predators capabilities (Dor, Valle‐Mora, Rodríguez‐Rodríguez, & Liedo, 2014; Hendrichs & Hendrichs, 1998; Hendrichs, Wornoayporn, Katsoyannos, & Hendrichs, 2007; Rao, Aguilar‐Argüello, Montoya, & Díaz‐Fleischer, 2014) or even the selection of complex and diffuse characteristics such as sexual competitiveness (McInnis, Shelly et al., 2002; Quintero‐Fong et al., 2016; Sánchez‐Rosario, Pérez‐Staples, Toledo, Valle‐Mora, & Liedo, 2017).

Although the desiccation resistant selected strain presents several advantages for field application, other relevant biological traits will have to been considered before their generalized use in the control program. A small‐scale demography evaluation of the selected strains points to an increased development time as a correlated response to selection (Tejeda et al., 2016). If this trait remains constant once the strain are scaled up to mass rearing, the longer development time could imply either the increase on rearing rooms capacity or the decrease of insect quantity produced by the biofactory. Although is highly probable that the field benefits of resistance strains outweigh the production costs (Barclay, 2005; Mumford, 2005), it will be important to gather information about their performance under mass production. Similarly, other relevant operative parameters such as dispersal and field survival (Flores et al., 2015), or their ability to inhibit female remating (Pérez Staples et al., 2017), will be useful to assertively determine the technological value of desiccation strains for the pest management program.

Finally, mass rearing processes enclose selection pressures that promotes high fertility, early sexual maturation and short life‐cycle times, which impacts insect life history (Cayol, 1999; Hernández, Toledo, Artiaga‐López, & Flores, 2009; Miyatake, 1998; Sgrò & Partridge, 2000). Consequently, other life history traits could decrease, as it happens in the case of tolerance to natural environment stressors (Hoffmann et al., 2001; Weldon et al., 2013) that reduce the performance of sterile insects. Artificial directional selection can be used to restore those characters lost during the mass rearing process. Furthermore, the highly controlled environments of laboratories and biofactories offer a unique opportunity to modify the resource and time constrains that shapes the evolution of life histories. This advantage, together with a strategically crossbreeding, make possible the production of phenotypes that even surpass the performance of wild individuals for a desirable trait (Folk & Bradley, 2005; Garland & Rose, 2009). Some evidences of the technical value of artificial selection as a strategy to enhance the efficiency of SIT have been performed in mass‐reared strains of *Ceratitis capitata* [Wiedemann] (McInnis, Shelly et al., 2002), *Cochliomyia hominivorax* [Coquerel] (Baumhover & Spates, 1965), *B. cucurbitae* [Coquillett] (Miyatake, 1996) and *A. ludens* [Loew] (Quintero‐Fong et al., 2016; Sánchez‐Rosario et al., 2017; Tejeda et al., 2016). Furthermore, the understanding of genetic mechanisms on specific characters surely would improve the use of artificial selection as a tool to improve the quality of mass‐reared insects used in pest management programs, especially those that incorporate the massive releases of sterile insects or parasitoids (Lommen et al., 2017).

## DATA ARCHIVING

Data for this study are available at the Dryad Digital Repository: https://doi.org/10.5061/dryad.r1s25
